# Supplementary material for: The Systems Biology Research Tool: evolvable open-source software
Source: BMC Syst Biol. 2008 Jun 29;2:55. doi: 10.1186/1752-0509-2-55 (PMC2446383; doi:10.1186/1752-0509-2-55)
Supplement: Additional file 1 — SBRT Archive. An archive of the current version of the Systems Biology Research Tool. [file 1752-0509-2-55-S1.zip › sbrt-1.4.0/doc/developers_guide/api/sbrt/kernel/math/algebra/Matrix.html]

Matrix


|  |  |  |
| --- | --- | --- |
| **Overview** \| **Package** \| **Class** \| **Use** \| **Tree** \| **Deprecated** \| **Index** \| **Help** | |  |
| **PREV CLASS**   **NEXT CLASS** | **FRAMES**    **NO FRAMES**     **All Classes** |  |
| SUMMARY: NESTED \| FIELD \| CONSTR \| METHOD | DETAIL: FIELD \| CONSTR \| METHOD |  |


---


## sbrt.kernel.math.algebra Interface Matrix<R,C,E>

**Type Parameters:**: `R` - the row index type.: `C` - the column index type.: `E` - the element type.

**All Known Subinterfaces:**: DoubleMatrix<R,C>, DoubleMatrixBuilder<R,C>, MatrixBuilder<R,C,E>, SmdMatrix<R,C>, SparseDoubleMatrix<R,C>

**All Known Implementing Classes:**: AbstractMdMatrix, AbstractSmdMatrix, DoubleMatrixWrap, HashMatrix, MatrixWrap, RowOrTreeMatrix, SparseDoubleMatrixWrap, TreeMatrix

---

``` public interface Matrix<R,C,E> ```

This interface is used to represent matrices. A matrix is
composed of rows and columns, which are referred to by
their corresponding indices. Typically these indices are
integer values from 1 to *m* and *n*, where
*m* is the number of rows and *n* is the number
of columns. In this matrix representation, however, indices
can be any type of `Object`. Indices are compared
via their Object.equals(Object) method.

**Author:**
:   This interface was written and documented by
    Jeremiah Wright while in the Wagner lab.

---

| **Method Summary** | |
| --- | --- |
| `int` | `columns()`             Returns the number of columns in this matrix. |
| `boolean` | `equals(java.lang.Object obj)`             Indicates if the provided object is equal to this matrix. |
| `Matrix<R,C,E>` | `getColumn(java.lang.Object columnIndex)`             Returns the column corresponding to the provided column index. |
| `java.util.Set<C>` | `getColumnIndices()`             Returns the set of column indices contained in this matrix. |
| `E` | `getElement(java.lang.Object rowIndex, java.lang.Object columnIndex)`             Returns the element of this matrix at the provided row and column indices. |
| `Matrix<R,C,E>` | `getRow(java.lang.Object rowIndex)`             Returns the row corresponding to the provided row index. |
| `java.util.Set<R>` | `getRowIndices()`             Returns the set of row indices contained in this matrix. |
| `int` | `hashCode()`             Returns a content-based hash code for this matrix. |
| `boolean` | `isColumnIndex(java.lang.Object obj)`             Indicates if the provided object is a column index in this matrix. |
| `boolean` | `isEmpty()`             Indicates if this matrix is empty. |
| `boolean` | `isRowIndex(java.lang.Object obj)`             Indicates if the provided object is a row index in this matrix. |
| `int` | `rows()`             Returns the number of rows in this matrix. |
| `java.lang.String` | `toString()`             Returns a string representation of this matrix. |

**Method Detail**

### getElement

```
E getElement(java.lang.Object rowIndex,
             java.lang.Object columnIndex)
```

:   Returns the element of this matrix at the provided
    row and column indices.

    :   **Parameters:**: `rowIndex` - the row index.: `columnIndex` - the column index. **Returns:**: the element of this matrix at the provided row and column indices.

---


### getColumnIndices

```
java.util.Set<C> getColumnIndices()
```

:   Returns the set of column indices contained in this
    matrix.

    :   **Returns:**: the set of column indices contained in this matrix.

---


### getRowIndices

```
java.util.Set<R> getRowIndices()
```

:   Returns the set of row indices contained in this
    matrix.

    :   **Returns:**: the set of row indices contained in this matrix.

---


### isRowIndex

```
boolean isRowIndex(java.lang.Object obj)
```

:   Indicates if the provided object is a row index in
    this matrix.

    :   **Parameters:**: `obj` - the potential row index. **Returns:**: `true` if the provided object is a row index in this matrix; `false` otherwise.

---


### isColumnIndex

```
boolean isColumnIndex(java.lang.Object obj)
```

:   Indicates if the provided object is a column index in
    this matrix.

    :   **Parameters:**: `obj` - the potential column index. **Returns:**: `true` if the provided object is a column index in this matrix; `false` otherwise.

---


### getRow

```
Matrix<R,C,E> getRow(java.lang.Object rowIndex)
```

:   Returns the row corresponding to the provided row index.
    A row is itself a 1 x n matrix, where n is the number of
    columns in this matrix.

    :   **Parameters:**: `rowIndex` - the index of the row to be returned. **Returns:**: the row corresponding to the provided row index.

---


### getColumn

```
Matrix<R,C,E> getColumn(java.lang.Object columnIndex)
```

:   Returns the column corresponding to the provided column index.
    A column is itself an m x 1 matrix, where m is the number of
    rows in this matrix.

    :   **Parameters:**: `columnIndex` - the index of the column to be returned. **Returns:**: the column corresponding to the provided column index.

---


### rows

```
int rows()
```

:   Returns the number of rows in this matrix.

    :   **Returns:**: the number of rows in this matrix.

---


### columns

```
int columns()
```

:   Returns the number of columns in this matrix.

    :   **Returns:**: the number of columns in this matrix.

---


### isEmpty

```
boolean isEmpty()
```

:   Indicates if this matrix is empty.

    :   **Returns:**: `true` if at least one row and column exists in this matrix; `false` otherwise.

---


### toString

```
java.lang.String toString()
```

:   Returns a string representation of this matrix.

    :   **Overrides:**: `toString` in class `java.lang.Object`
    :   **Returns:**: a string representation of this matrix.

---


### equals

```
boolean equals(java.lang.Object obj)
```

:   Indicates if the provided object is equal to this matrix.
    Two matrices are considered equal if they share the same
    sets of column and row indices and the elements in each
    matrix for each column-row index pair are the same.

    :   **Overrides:**: `equals` in class `java.lang.Object`
    :   **Parameters:**: `obj` - the object with which to compare. **Returns:**: `true` if this object equals the provided object; `false` otherwise.

---


### hashCode

```
int hashCode()
```

:   Returns a content-based hash code for this matrix.

    :   **Overrides:**: `hashCode` in class `java.lang.Object`
    :   **Returns:**: a content-based hash code for this matrix.


---


|  |  |  |
| --- | --- | --- |
| **Overview** \| **Package** \| **Class** \| **Use** \| **Tree** \| **Deprecated** \| **Index** \| **Help** | |  |
| **PREV CLASS**   **NEXT CLASS** | **FRAMES**    **NO FRAMES**     **All Classes** |  |
| SUMMARY: NESTED \| FIELD \| CONSTR \| METHOD | DETAIL: FIELD \| CONSTR \| METHOD |  |


---
